# Tooth brushing behavior and oral health care of people with early childhood autism in Germany

**DOI:** 10.1007/s00784-025-06194-8

**Published:** 2025-02-05

**Authors:** H. Kraus, A. G. Schulte, O. Fricke, Peter Schmidt

**Affiliations:** 1 Department of Special Care Dentistry, Witten/Herdecke University, Alfred-Herrhausen-Straße 50, 58455 Witten, Germany; 2 Faculty of Health, Department of Medicine, Witten/Herdecke University, Alfred-Herrhausen- Straße 50, 58455 Witten, Germany; 3 Department of Child and Adolescent Psychiatry and Psychotherapy, Klinikum Stuttgart, Prießnitzweg 24, 70174 Stuttgart, Germany

**Keywords:** Autism spectrum disorders, Special care patients, Dentistry, Oral health, Mental health

## Abstract

**Objectives:**

Only a little is known about the tooth brushing behaviors and oral health care of people with early childhood autism (P-EA). To remedy this, a survey was carried out with P-EA of all ages.

**Materials and methods:**

In summer 2021, an online survey questionnaire with 124 questions, addressed only at caregivers of P-EA, was sent to all members of the Bundesverband Autismus Deutschland e.V. Our present study evaluated the 20 survey questions related to dental self-care.

**Results:**

In total, 130 questionnaires were evaluated. The mean age of the P-EA was 22.7 years (min: 3y; max: 60y; SD ± 14.1y). Only 17.7% (*n* =23) of the P-EA were female, while 82.3%(*n* =107) were male. Overall, 54.6% (*n* = 71) of P-EA are always actively assisted in tooth brushing. Although 57.7% (*n* = 75) of P-EA brushed teeth twice daily, 39.2% brushed for one to two minutes (*n* = 51). P-EA younger than 18 years old selected toothpaste primarily for taste reasons (32.7%;*n* = 18). Only 9.3% (*n* = 7) of adult P-EA selected toothpaste because of the fluoride content. Manual toothbrushes were preferred by 40.8% (*n* = 53) of the P-EA, and electric toothbrushes by 34.6% (*n* = 45). Almost three quarters (71.5%;*n* = 93) did not use additional dental care implements. The same number (75.4%;*n* = 98) of P-EA had not practiced supervised brushing in a dental office.

**Conclusions/Clinical relevance:**

Active support in dental self-care is crucial for a majority of the P-EA in Germany, regardless of their age. Professional support needs to be fundamentally improved. Clinical concepts for practical instruction in dental hygiene should be developed for P-EA.

## Introduction

According to official registration records, Germany had a population of 83.2 million people in 2021 [1]. At around the same time, in 2020, approximately 7.9 million of these inhabitants were people with severe disability. This community, which includes people with autism spectrum disorder (ASD), thus constitutes 9.5% of the German population [1]. Until fairly recently, ASD was referred to as “autism disorder.” Now, both the DSM-5 (Diagnostic and Statistical Manual of Mental Disorders-5) nosography–the dominant psychiatric classification system used in the United States– by the American Psychiatric Association’s (APA) and the World Health Organization’s (WHO) ICD-11 classification system, which was newly released early in 2022, use the umbrella term “autism spectrum disorder” to describe this set of conditions. The inclusion of the term “spectrum” reflects the heterogeneous presentation of autism and the flowing boundaries between the sub-types of this pervasive and complex neurodevelopmental condition [2]. Previously, the ICD-10 system distinguished three clinical sub-types of autism: Early childhood autism (F84.0), Atypical autism (F84.1), and Asperger syndrome (F84.5) [3].

Epidemiological studies on the prevalence of autism suggest that there has been a steady increase in diagnoses since the 1990s [4, 5]. Maenner et al., for example, report a prevalence rate of 14.6 per 1000 people, or of 0.5–1% of the population, for ASD or, expressed differently, that one in 68 children show behaviors typical for ASD [6]. Furthermore, according to a recent study conducted by the Autism and Developmental Disabilities Monitoring Network, one in 44 children in the USA and Canada is diagnosed with ASD. In terms of prevalence, this corresponds to 23 children with ASD per 1000 children [7]. Similar prevalence rates are reported for countries such as Japan and South Korea [8]. Considering these numbers, the probability that dentists will at some point in their careers encounter patients with autism is accordingly high.

Early childhood autism (EA), also known as Kanner autism, was first described in 1943 by the psychiatrist Leo Kanner. This severe developmental disorder is characterized by complications with language development and ensuing verbal communication problems as well as difficulties with social interactions and relationships. People with early childhood autism (P-EA) also feature repetitive stereotypical patterns of behavior and a desire for the maintenance of specific routines. In most cases, disruption of such routines is reported to elicit stress or fear in P-EA. Moreover, EA is often accompanied by severe intellectual impairment [9, 10, 11]. Kanner also observed that ASD was diagnosed three to four times more commonly in boys than in girls [9].

In the context of access to dental health care for P-EA, it is extremely important to emphasize that the various behavioral characteristics for this manifestation of autism may greatly affect a dentist’s ability to provide dental treatments. The need to accommodate the specific behaviors, described above, of dental patients with EA may, therefore, be quite challenging for dentists, who must also be capable of dealing with the social aspects of the situation. For example, deviation from a familiar routine during dental treatment may elicit fear in P-EA, a response that can complicate or even entirely prevent the provision of a dental treatment [9, 11, 12]. Because intellectual impairment is frequently a diagnostic feature of EA, communication barriers may further compound the encountered challenges. Additionally, depending on the severity of intellectual impairment, epileptic seizures are known to occur in P-EA [13]. Because these are associated with an increased risk for falls and, hence, with an increased risk for oro-facial injuries [14], this aspect is also highly pertinent to dental health care.

In view of the specific characteristics and comorbidities that are known to be associated with EA, we believe that it is expedient to still differentiate sub-groups within the autism spectrum according to the ICD-10 criteria in the context of research. In our opinion, this allows better identification and accommodation of specific needs for autism subtypes, such as EA. In this study, we therefore explicitly looked at the dental care status of P-EA, as defined by ICD-10.

Apart from data published in three international studies in the last decade from Brazil [15], England [16], and Jordan [17], the authors of the current study are not aware of any other published data related exclusively to the dental and oral self-care of P-EA. In addition, there is only little data available on how satisfied family members and caregivers with the quality of dental care for P-EA are, or on the specific needs this group of dental patients may have. A further problem in this regard is the identification of suitable dental practices that are barrier-free, not only in terms of mobility, but also in terms of the empathetic capacity of the dentists and their teams working there. In two previous studies on special care dentistry, almost 90% of the surveyed dentists reported that they had not been adequately educated regarding to the specific characteristics of persons with disabilities, nor had they been adequately trained to treat these patient groups. Furthermore, a wide-spread wish for greater instruction in special care dentistry in dental school [18, 19] was expressed in these studies.

To be able to address the needs of P-EA more specifically, and thus improve not only the quality of dental care and prophylaxis in this community, but also their life quality, it is essential to investigate the needs of this sub-group of autists extensively. To this end, a comprehensive survey was conducted. The present study set out to evaluate the results for the sub-section of the survey that was related to oral self-care and supportive dental health care of P-EA.

## Materials and methods

A nationwide, epidemiological, cross-sectional study, limited to P-EA, was conducted in cooperation with the Bundesverband Autismus Deutschland e.V. (German Association for Autism), the federal German umbrella association for autism, using an online-questionnaire (SoSci-Survey GmbH, München, Germany). According to the federal association’s own report, they had 10,812 members during the survey period in the summer (15th June until 15th September) of 2021. With the help of the federal association, data regarding to dental health care for P-EA were collected from all German federal states.

Information about participation in the study was made available on the website of the German Association for Autism, for the three-month survey period during the summer of 2021. In addition, an informative article and a flyer were posted on the website. According to information provided by the policy officer (F. D.) of the German Association for Autism, the link to the questionnaire was distributed to 4299 e-mail addresses via the association’s mail distribution list. This distribution list comprised the addresses of lower-tier national and regional autism associations and private e-mail addresses. The national and regional associations, in turn, forwarded the link to their members. To improve the return rate, the policy officer (F.D.) sent two reminders within the three-month survey period via distribution list, one after the first survey month and one after the second survey month.

For the purpose of the comprehensive online survey, the research team designed a questionnaire comprising 124 questions, which were largely related to the behaviors of P-EA in professional dental settings and their regular oral and dental self-care behaviors at home. There were also questions on how satisfied caregivers were with the quality of dental health care their dependents with EA received and, additionally, questions requesting standard medical and sociodemographic information. To gain as great a wealth of pertinent information as possible, a mixed questionnaire was compiled, comprising close-ended (*n* = 54), open-ended (*n* = 6), and mixed questions, with closed and open responses (*n* = 64). The questionnaire was exclusively directed at P-EA (ICD-10: F84.0) and was to be filled out by family members, legal representatives, or primary caregivers of P-EA.

At the beginning of the questionnaire, the first question asked for the autism subtype. This ensured that only questionnaires in which the participants stated that their family member had a diagnosis of ‘early childhood autism’ (ICD-10: F84.0) were included.

In the following, for the sake of simplicity, the parents or other persons who completed the questionnaires for a dependent with EA will be referred to as caregivers, as will be all other persons supporting or supervising an autist with EA. These terms have been used similarly in other publications by the authors. Further explanations and information in this respect may be found there [20].

Consequently, the questionnaire used in the present study is based on the questionnaire used by Schmidt et al. in the study regarding to the oral health of people with Down syndrome in Germany [20]. This questionnaire was adapted to the needs/specialities of the group of P-EA in suitable and necessary parts and the items were evaluated. Because validation should always be carried out when designing and creating new questionnaires, this was done in the form of ‘content validation’ with regard to the present questionnaire for P-EA. This serves to check that the test items are constructed and selected in the course of operationalisation in such a way that they represent the characteristic of interest and that the characteristics to be measured are actually recorded with the items used [21]. Existing theories, research findings and items were therefore used in the design of this questionnaire. The content of the questionnaire was then reviewed by a group of experts in various rounds of consultation. The content validation did not include the implementation of statistical procedures for validation [21, 22]. In addition, a pre-test of the online questionnaire was subsequently carried out by members of the study team, other dentists and people from the German Association for Autism. This included, among other things, a section for each question with the option for respondents to provide feedback and comments. In a final consultation with the group of experts, the feedback received in the pretest was then taken into the final version of the questionnaire.

The online survey was anonymous, and the responses were collected in compliance with data protection laws. The questionnaire could also be requested in paper and pencil format from the federal autism association. Only fully completed questionnaires, with responses to every question, were included in the study. Questionnaires that were only started, but not completed, were excluded. This information applies to the entire study and this publication.

A positive vote from the ethics committee of the Witten/Herdecke University had been obtained prior to the study (#82/2021).

The present study, which was conducted within the framework of the comprehensive survey, investigated the results for a sub-section of the survey questions (20 questions) related to the oral health self-care of P-EA. The following aspects were investigated in this paper: sociodemographic information about the P-EA and the person who completed the questionnaire (7 questions), tooth brushing behavior (6 questions) level of assistance with oral self-care at home (2 questions), use of caries-preventive fluoride products at home (2 questions) and professional dental health care and support (3 questions).

The data from the included questionnaires were transferred to Microsoft Excel 2016 (Microsoft Corporation, Redmond, Washington, DC, USA). For this purpose, the data were categorized into different subgroups which also took the sociodemographic parameters “sex” and “age group” into account. The data were statistically analyzed using “Microsoft Excel 2016” and “IPM SPSS Version 26” (IBM Corporation, New York, NY, USA) and described via relative and absolute frequencies, mean values, minimum values, maximum values, and standard deviation. The Chi-square test was used to test the statistical significance of correlations and differences between the various subgroups, which were also categorized for sex (male, female) and age (< 18 years old, ≥ 18 years old).

## Results

### Sociodemographic data

One-hundred and thirty (130) fully completed questionnaires with responses to all questions, could be included in our study. In percentage terms, this corresponds to 39.4% of all questionnaires that had been begun. The other 60.6% of the questionnaires were excluded. For almost half of the excluded questionnaires, this was due to the fact, that the participants already stated in the first question that their family member did not have a diagnosis for ‘early childhood autism’ (ICD-10: F84.0). Others were excluded because participants had to quit the questionnaire, either already at the level of the privacy notice, or at some later point, without having completed the questionnaire.

Of the 130 surveyed P-EA, 107 (82.3%) were male and 23 (17.7%) were female. The mean age of all included persons was 22.7 years (SD ± 14.1).

A total of 66 (50.8%) of the P-EA had a legal representative or guardian; 54 (41.5%) did not have a legal representative or guardian because they were not 18 years old yet and their parents still had custody; only 10 (7.7%) were without legal representative or guardian. 82 (63.1%) of the P-EA lived with their parents; 38 (29.2%) were in supervised living homes, and two (1.5%) lived on their own (Table 1).

**Table 1:** Sociodemographic data of people with early childhood autism (P-EA) reported by those who completed the questionnaire

People with early childhood autism		
**Sex**	*n*	%
Male P-EA	107	82.3
Female P-EA	23	17.7
**Age (in years)**		
Mean (all P-EA) ± SD	22.7 ± 14.1	
Median (all P-EA)	19	
Range (all P-EA, *n* = 130)	3–60	
Mean (all male P-EA) ± SD	21.8 ± 13.6	
Median (all male P-EA)	19	
Range (all male P-EA, *n* = 107)	3–60	
Mean (all female P-EA) ± SD	26.7 ± 15.8	
Median (all female P-EA)	21	
Range (all female P-EA, *n* = 23)	4–56	
Mean (all < 18 years old P-EA) ± SD	10.7 ± 4.0	
Median (all < 18 years old P-EA)	12	
Range (all < 18 years old P-EA, *n* = 55)	3–17	
Mean (male < 18 years old P-EA) ± SD	10.5 ± 4.0	
Median (male < 18 years old P-EA)	11.5	
Range (male < 18 years old P-EA, *n* = 46)	3–17	
Mean (female < 18 years old P-EA) ± SD	11.8 ± 3.9	
Median (female < 18 years old P-EA)	12	
Range (female < 18 years old P-EA, *n* = 9)	4–16	
Mean (all ≥ 18 years old P-EA) ± SD	31.5 ± 12.4	
Median (all ≥ 18 years old P-EA)	28	
Range (all ≥ 18 years old P-EA, *n* = 75)	18–60	
Mean (male ≥ 18 years old P-EA) ± SD	30.4 ± 11.9	
Median (male ≥ 18 years old P-EA)	26	
Range (male ≥ 18 years old P-EA, *n* = 61)	18–60	
Mean (female ≥ 18 years old P-EA) ± SD	36.4 ± 13.2	
Median (female ≥ 18 years old P-EA)	35.5	
Range (female ≥ 18 years old P-EA, *n* = 14)	18–56	
**Legal guardian**	**n**	**%**
Has a legal guardian	66	50.8
Has no legal guardian because the person with EA is under 18 years old and the parents have the custody	54	41.5
Has no legal guardian	10	7.7
**Living situation**	**n**	**%**
Alone	2	1.5
At the parents‘ home	82	63.1
At a family member’s home	2	1.5
In supervised living	38	29.2
Other *(free text specification)*	6	4.6

In 76.9% (*n* = 100) of the cases, the questionnaires had been completed by a parent and in 10.8% (*n* = 14) by a primary caregiver. The remaining questionnaires had been completed by another family member, a legal representative, or other caregiver. For P-EA younger than 18 years, the questionnaire had been completed by a parent in 94.5% (*n* = 52) of the cases; for P-EA older than 18 years, the questionnaires had been completed by a parent in 64.0% (*n* = 48) (Table 2).

**Table 2:** Characteristics of the persons who completed the questionnaire of the people with early childhood autism (P-EA)

Characteristic of the people who completed the questionnaire		
**Who completed the questionnaire? (For all P-EA, n = 130)**	*n*	%
One parent	100	76.9
Both parents	2	1.5
A family member (non-parent)	3	2.3
A legal guardian	3	2.3
A caregiver	14	10.8
Other *(free text response)*	6	4.6
No statement	2	1.5
**Age (in years)**		
Mean ± SD Median Range (*n* = 124 (*n* = 6 no statement))	50.9 ± 12.4 50.0 25–94	
**Who completed the questionnaire? (For all < 18 years old P-EA**,**n = 55)**	**n**	**%**
One parent	52	94.5
Both parents	0	0
A family member (non-parent)	0	0
A legal guardian	0	0
A caregiver	0	0
Other *(free text response)*	3	5.5
No statement	0	0
**Age (in years)**		
Mean ± SD Median Range (*n* = 55 (*n* = 1 no statement))	45.9 ± 7.5 45.0 32–83	
**Who completed the questionnaire? (For all ≥ 18 years old P-EA, n = 75)**	**n**	**%**
One parent	48	64.0
Both parents	2	2.7
A family member (non-parent)	3	4.0
A legal guardian	3	4.0
A caregiver	14	18.7
Other *(free text response)*	3	4.0
No statement	2	2.7
**Age (in years)**		
Mean ± SD Median Range (*n* = 70 (*n* = 5 no statement))	54.7 ± 14.0 55.0 25–94	

The data was collected from all German federal states. During the survey period in 2021, the total population in Germany numbered 83.2 million people [23]. In Figure the proportional distribution of the study participants in relation to the number of inhabitants in the respective German federal states is shown. The percentage distribution of participants corresponded with the proportional distribution of inhabitants per federal state. In this context, the comparatively high participation rate of 25.4% for the federal state of Lower Saxony stood out noticeably.

**Fig. 1:**
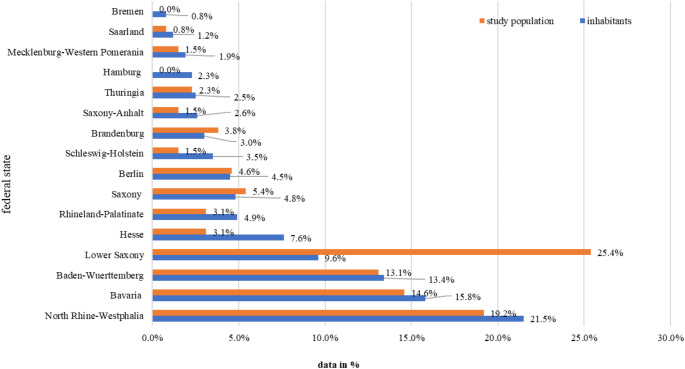
Comparison of the percentage distribution of inhabitants per German federal state in 2021 (blue), for a total population of 83.2 million people, with the percentage distribution of the study population per federal state (orange), for a total of 130 participants

### Tooth brushing behavior and assisted oral health care

Table 3 shows the general tooth brushing preferences, the dental care at home and the use of caries-preventive fluoride products for all P-EA (all: *n* = 130). They were also categorized by sex (male: *n* = 107; female; *n* = 23) and age (< 18 years: *n* = 55; ≥ 18 years: *n* = 75).

All in all, 54.6% (*n* = 71) of P-EA need to be actively assisted with tooth brushing. Only 13.8% (*n* = 18) always brush their teeth on their own. Although a difference was found in the comparison of those who brushed alone (male: 15.9% (*n* = 17); female: 4.3% (*n* = 1)) and those who were actively assisted (male: 55.1% (*n* = 59); female: 52.2% (*n* = 12)); the difference between the genders was not statistically significant (*p* = 0.217) (Table 3).

Among the surveyed P-EA, 59.8% (*n* = 67) were, as a rule, always actively assisted by the same person, whereas 37.5% (*n* = 42) were generally assisted by several persons (Table 3).

In respect to toothbrush preferences, 40.8% (*n* = 53) of the P-EA used manual toothbrushes, while 34.6% (*n* = 45) used an electric toothbrush. Percentage differences also emerged here between sexes: 43.9% (*n* = 47) of the surveyed male P-EA and 26.1% (*n* = 6) of the female P-EA used manual toothbrushes in comparison to 32.7% (*n* = 35) of the male P-EA and 43.5% (*n* = 10) of the female P-EA who used electric toothbrushes. This difference between the genders, however, was also not significant (*p* = 0.414). Furthermore, the association between the person’s age and the type of preferred toothbrush, i.e., manual or electric, was not significant (*p* = 0.122). Only very few P-EA alternated between using manual and electric toothbrushes (14.6%; *n* = 19) (Table 3).

In regard to toothpaste preferences, 50.8% (*n* = 66) of the P-EA used almost all types of toothpaste available on the German market. The association between age groups and the use of almost all types of toothpaste versus the use of only a few types of toothpaste was not significant (*p* = 0.492). In the age group younger than 18 years, 41.8% (*n* = 23) of the P-EA used almost all types of toothpaste, while 18.2% used only a few types. In the age group older than 18 years, 57.3% (*n* = 43) of the P-EA used all types of toothpaste, while 12.0% used only a few types (Table 3).

In the sub-group “All P-EA,” 71.5% (*n* = 93) did not use dental hygiene implements other than toothbrushes. This percentage was found to be similar for all of the sub-groups (male: 71.0%, *n* = 76; female: 73.9%, *n* = 17; under 18 years old: 69.1%, *n* = 38; over 18 years old: 73.3%, *n* = 55).

Preferences for other types of toothpaste (10.0%, *n* = 13) or dental hygiene products, e.g., specifically requested children’s toothpaste, or the type of toothpaste used by their parents (also 10.0%, *n* = 13), were also observed (Table 3).

In regard to tooth brushing length and frequency, it emerged that 39.2% (*n* = 51) of P-EA spend only one to two minutes in brushing their teeth. On the other hand, 57.7% (*n* = 75) of all P-EA brushed their teeth twice daily. A prominent observation in this respect was that while only 53.3% (*n* = 57) of male autists with EA brushed twice daily, and 18.7% (*n* = 20) brushed their teeth three times daily, 78.3% (*n* = 18) of female autists with EA brushed their teeth twice daily, and 8.7% (*n* = 2) brushed three times daily. Lastly, this difference between the genders, did not, however, prove significant (*p* = 0.340).

The comparison of age group and frequency of daily tooth-brushing, however, reveal a notable percentage divergence between age groups: While 60.0% (*n* = 33) of P-EA younger 18 years brushed their teeth twice daily, and 9.1% (*n* = 5) brushed three times daily, 56.0% (*n* = 42) of the P-EA in the older than 18 years group, brushed twice daily, and 22.7% (*n* = 17) brushed three times daily. Nonetheless, with a p-value of *p* = 0.302, the difference in tooth brushing frequency between the age groups was not significant (Table 3).

Based on the responses provided by the caregivers who help the P-EA brush their teeth, 42.3% (*n* = 55) of the P-EA began tooth brushing in their first year of life. A notable finding was that only 28.0% (*n* = 21) of the adult autists with EA had begun tooth brushing in their first year of life (Table 3).

**Table 3:** Information about the tooth brushing preferences and oral health care of people with early childhood autism (P-EA), as reported by those who completed the questionnaire

		Sex		Age	
	All	Male	Female	< 18 years	≥ 18 years
	(*n* = 130)	(*n* = 107)	(*n* = 23)	(*n* = 55)	(*n* = 75)
Does the person with EA receive assistance in tooth brushing? * ^**2**^					
always brushes teeth alone	13.8% (18)	*15.9% (17)*	*4.3% (1)*	7.3% (4)	18.7% (14)
always needs assistance with tooth brushing	54.6% (71)	*55.1% (59)*	*52.2% (12)*	60.0% (33)	50.7% (38)
		*p*=0.329	*p*=0.329		
always needs instructions for tooth brushing	10.8% (14)	11.2% (12)	8.7% (2)	12.7% (7)	9.3% (7)
receives assistance with tooth brushing at least once a week	5.4% (7)	4.7% (5)	8.7% (2)	3.6% (2)	6.7% (5)
receives assistance with tooth brushing at least once a day	15.4% (20)	13.1% (14)	26.1% (6)	16.4% (11)	14.7% (11)
Who assists with tooth brushing? * ^3^					
usually the same person	59.8% (67)	62.6% (57)	47.6% (10)	76.5% (39)	45.9% (28)
almost always several persons	37.5% (42)	34.1% (31)	52.4% (11)	23.5% (12)	49.2% (30)
does not apply	2.7% (3)	3.3% (3)	0.0% (0)	0.0% (0)	4.9% (3)
How often does the person with EA usually bush teeth? * ^2^					
teeth cannot be brushed regularly	4.6% (6)	5.6% (6)	0.0% (0)	5.5% (3)	4.0% (3)
once a day	18.5% (24)	19.6% (21)	13.0% (3)	23.6% (13)	14.7% (11)
twice a day	57.7% (75)	*53.3% (57)*	*78.3% (18)*	*60*,*.0% (33)*	*56.0% (42)*
three times a day	16.9% (22)	*18.7% (20)*	*8.7% (2)*	*9.1% (5)*	*22.7% (17)*
		*p* = 0.340	*p* = 0.340	*p* = 0.302	*p* = 0.302
after every meal	2.3% (3)	2.8% (3)	0.0% (0)	1.8% (1)	2.7% (2)
**How long does the person with EA usually accept tooth brushing?**					
less than 1 min	25.4% (33)	25.2% (27)	26.1% (6)	27.3% (15)	24.0% (18)
1–2 min	39.2% (51)	41.1% (44)	30.4% (7)	41.8% (23)	27.3% (28)
2–3 min	22.3% (29)	21.5% (23)	26.1% (6)	18.2% (10)	25.3% (19)
different every time	13.1% (17)	12.1% (13)	17.4% (4)	12.7% (7)	13.3% (10)
What type of toothbrush does the person with EA use for tooth brushing? * ^2^					
manual toothbrush	40.8% (53)	*43.9% (47)*	*26.1% (6)*	*43.6% (24)*	*38.7% (29)*
electric toothbrush	34.6% (45)	*32.7% (35)*	*43.5% (10)*	*27.3% (15)*	*40.0% (30)*
		*p* = 0.414	*p* = 0.414	*p* = 0.122	*p* = 0.122
sonic electric toothbrush	7.7% (10)	6.5% (7)	13.0% (3)	7.3% (4)	8.0% (6)
both manual and electric toothbrush	14.6% (19)	14.0% (15)	17.4% (4)	21.8% (12)	9.3% (7)
other *(free text response)*	2.3% (3)	2.8% (3)	0.0% (0)	0.0% (0)	4.0% (3)
What type of toothpaste does the person with EA use for tooth brushing? * ^1^ * ^2^					
almost all types of toothpaste	50.8% (66)	51.4% (55)	47.8% (11)	*41.8% (23)*	*57.3% (43)*
only a few types of toothpaste	14.6% (19)	13.1% (14)	21.7% (5)	*18.2% (10)*	*12.0% (9)*
				*p* = 0.492	*p* = 0.492
the toothpaste should contain fluoride	15.4% (20)	15.0% (16)	17.4% (4)	23.6% (13)	9.3% (7)
the selection is mainly based on type	10.0% (13)	8.4% (9)	17.4% (4)	7.3% (4)	12.0% (9)
the selection is mainly based on ingredients	14.6% (19)	14.0% (15)	17.4% (4)	12.7% (7)	16.0% (12)
the selection is mainly based on taste	23.8% (31)	23.4% (25)	26.1% (6)	32.7% (18)	17.3% (13)
the selection is mainly based on price	0.8% (1)	0.9% (1)	0.0% (0)	1.8% (1)	0.0% (0)
other *(free text response)*	10.0% (13)	9.3% (10)	13.0% (3)	12.7% (7)	8.0% (6)
Are additional dental hygiene implements other than a toothbrush used by the person with EA? * ^1^					
Interdental brushes	8.5% (11)	7.5% (8)	13.0% (3)	3.6% (2)	12.0% (9)
Dental floss	16.9% (22)	16.8% (18)	17.4% (4)	20.0% (11)	14.7% (11)
Dental woods	1.5% (2)	1.9% (2)	0.0% (0)	0.0% (0)	2.7% (2)
Tongue cleaner	4.6% (6)	5.6% (6)	0.0% (0)	5.5% (3)	4.0% (3)
no additional implements	71.5% (93)	71.0% (76)	73.9% (17)	69.1% (38)	73.3% (55)
other *(free text response)*	10.0% (13)	11.2% (12)	4.3% (1)	12.7% (7)	8.0% (6)
**At what age did the person with EA start tooth brushing?**					
in the first year of life	42.3% (55)	41.1% (44)	47.8% (11)	61.8% (34)	28.0% (21)
at the age of 1 year	30.0% (39)	32.7% (35)	17.4% (4)	27.3% (15)	32.0% (24)
at the age of 2 years	6.9% (9)	7.5% (8)	4.3% (1)	7.3% (4)	6.7% (5)
at the age of 3 years	5.4% (7)	4.7% (5)	8.7% (2)	3.6% (2)	6.7% (5)
I/We do not remember (anymore)	15.4% (20)	14.0% (15)	21.7% (5)	0.0% (0)	26.7% (20)

*^1^ Multiple responses possible, *^2^ The p-values of the Chi-square test are shown in the following table. *^3^ The total number of all participants is *n* = 112 here, instead of *n* = 130, because *n* = 18 P-EA always brushed their teeth alone. The sum of individual frequencies in the groupings, therefore, diverges from the number for “n” in the column headings.

### Use of fluoride products in household

Various caries-preventive fluoride products are available for domestic use in Germany. In the present study, questions were asked about the use of three fluoride products that are available on the market (mouth rinses with fluoride, fluoride gels, and fluoridated table salt). In response to the questions on the use of fluoride, the majority of caregivers (60.8%, *n* = 79) answered that fluoridated table salt was used in the P-EA’s household. The use of fluoride gels (18.5%, *n* = 24) or fluoride mouth rinses (7.7%, *n* = 10) was considerably less widespread. Almost half of the P-EA had been given fluoride tablets as small children (Table 4). This applied to both sexes and both age groups.

**Table 4:** Information on the household use of caries-preventive fluoride products by people with early childhood autism (P-EA), as reported by those who completed the questionnaire

		Sex		Age	
	All (*n* = 130)	Male (*n* = 107)	Female (*n* = 23)	< 18 years (*n* = 55)	≥ 18 years (*n* = 75)
Are any caries-preventive fluoride products used by the person with EA? * ^3^					
• Mouth rinse with fluoride • Gels with fluoride • Table salt with fluoride • no use of these fluorides	7.7% (10) 18.5% (24) 60.8% (79) 16.9% (22)	8.4% (9) 17.8% (19) 65.4% (70) 16.9% (18)	4.3% (1) 21.7% (5) 39.1% (9) 17.4% (4)	9.1% (5) 29.1% (16) 74.5% (41) 18.2% (10)	6.7% (5) 10.7% (8) 50.7% (38) 16.0% (12)
**Did the person with EA receive fluoride tablets as a child?**					
• yes • no • I/We do not remember (anymore)	46.9% (61) 34.6% (45) 18.5% (24)	48.6% (52) 31.8% (34) 19.6% (21)	39.1% (9) 47.8% (11) 13.0% (3)	43.6% (24) 49.1% (27) 7.3% (4)	49.3% (37) 24.0% (18) 26.7% (20)

*^3^ In the questionnaire, three separate questions asked about the use of fluoride (fluoride in mouth rinse, fluoride in gels, fluoride in table salt) and summarized here. The sum of individual use of fluoride by the person with EA, therefore, diverges from the number for “n” in the column headings.

### Professional supportive oral health care

Independent of age and sex, almost half of the P-EA (46.9%, *n* = 61) had not been instructed in tooth brushing techniques or been shown how to brush their teeth at a dental office. Similarly, 43.8% (*n* = 57) of caregivers had not been shown how to brush the P-EA’s teeth and had not been instructed how to do so by a dental professional. Tooth brushing had also not been practiced at a dental practice with 75.4% (*n* = 98) of the P-EA (Table 5).

**Table 5:** Information about professional supportive dental health care for people with early childhood autism (P-EA), as reported by those who completed the questionnaire

		Sex		Age	
	All (*n* = 130)	Male (*n* = 107)	Female (*n* = 23)	< 18 years (*n* = 55)	≥ 18 years (*n* = 75)
**Were tooth brushing techniques explained and demonstrated to the person with EA at the dental practice?**					
• yes • no • other *(free text response)*	41.5% (54) 46.9% (61) 11.5% (15)	41.4% (44) 48.6% (52) 10.3% (11)	43.5% (10) 39.1% (9) 17.4% (4)	43.8% (24) 41.8% (23) 14.5% (8)	40.0% (30) 50.7% (38) 9.3% (7)
**Was tooth brushing practiced with the person with EA at the dental practice?**					
• yes • no • other *(free text response)*	18.5% (24) 75.4% (98) 6.2% (8)	20.6% (22) 73.8% (79) 5.6% (6)	8.7% (2) 82.6% (19) 8.7% (2)	23.6% (13) 70.9% (39) 5.5% (3)	14.7% (11) 78.7% (59) 6.7% (5)
**Were tooth brushing techniques explained and demonstrated to the caregiver of the person with EA at the dental practice?**					
• yes • no • does not apply • other *(free text response)*	40.8% (53) 43.8% (57) 10.0% (13) 5.4% (7)	43.9% (47) 42.1% (45) 9.3% (10) 4.7% (5)	26.1% (6) 52.2% (12) 13.0% (3) 8.7% (2)	43.6% (24) 49.1% (27) 3.6% (2) 3.6% (2)	38.7% (29) 40.0% (30) 14.7% (11) 6.7% (5)

## Discussion

Substantial information in regard to oral health care of P-EA could be gained in a comprehensive, nationwide survey. The present study, which was conducted in the context of this survey, evaluated 20 questions from the survey questionnaire in respect to the oral health care behaviors of P-EA. The epidemiological data from the survey permitted an assessment of the tooth brushing behavior, oral self-care measures at home and professional oral health care support in dental practices for P-EA in Germany for the first time. Moreover, the survey data allowed investigation of these aspects for P-EA of all ages and both sexes, instead of only for predefined sample groups. In the last ten years, there have been only a few comparable international publications on oral and dental care for people with ASD to also take all age groups into account. These studies are from Brazil [15], England [16] and Jordan [17]. Quite frequently, studies on this topic focus on data for children and adolescents only [11, 24, 25, 26, 27, 28, 29, 30, 31, 32]. To our knowledge, there is no published data from Germany exclusively on the tooth-brushing behaviors and oral care of people with ASD, let alone data for an autism sub-group such as P-EA.

For our study, we could evaluate a total of 130 questionnaires that had been fully completed, with responses to every question. At first, this return rate appears quite poor, in view of the 4299 e-mails that had been forwarded to the state and the regional autism associations and to individual members of the German Association for Autism. However, in a debriefing session with the policy officer (F.D.) of this association, several limiting factors could be identified. For one, the federal autism association, as the primary umbrella organization had no means of establishing if all the e-mail addresses in their mail distribution list were still active at the time of the survey. Secondly, it could not be ruled out that some state or regional association had failed, in turn, reliably forwarded the e-mail to all of their members, in a “top-down” procedure. In addition, the distributed questionnaire was exclusively directed at persons who had been diagnosed with EA (ICD-10: F84.0). Because the federal association did not possess information about the percentage distribution of their members across autism sub-groups, we could not calculate the return rate in respect to the EA group.

Another factor affecting the return rate may have been the timing of the survey, which was conducted during the summer months of June, July, August, and September 2021, at a time when many people resumed travelling or going on holiday after the Covid-19-related lockdown in the summer of 2020 [33]. A second time-related aspect may have been that, at the time of the survey, in 2021, the diagnostic classification of autism was not yet uniformly regulated around the globe. Although the WHO’s ICD-11 system was certainly already known in some specialist groups and associations, it only came into force in January 2022. As a result, the different manifestations of autism are no longer officially described/classified as clinical subtypes (as in the ICD-10 system) until this date (January 2022). The ICD-11 now classifies the different manifestations of autism together under the umbrella term “autism spectrum disorder” (ASD). Against this backdrop, the fact that participation in the survey was limited to P-EA may have caused some caregivers to feel uncertain about whether or not the survey applied to them. This may also have contributed to the comparatively low number of participants.

Despite acknowledging the heterogeneity of autism and the flowing boundaries between autism types, we, nonetheless, believe that it remains crucially important to distinguish between sub-types of ASD, particularly in research related to special care dentistry. From our vantage, there is a noticeable difference, for instance, between the tooth brushing capabilities of autists formerly classified as P-EA by the ICD-10 system and those of autists from other former classification groups, e.g., Asperger syndrome. The observation that there are differing incidences of various comorbidities in autism subgroups further illustrates that substantial differences exist between the groups [34]. Because differences such as these can affect the provision of dental treatment in manifold ways, it would be helpful to dentists to be aware of autism sub-group characteristics so that they can prepare specifically to accommodate the needs of dental patients from these groups.

In this context, it deserves mention that numerous caregivers of P-EA left positive feedback in regard to the survey in the open response section. The respondents in particular emphasized how important topics in regard to the oral and dental care of P-EA were to them, particularly in light of the considerable effort involved in providing oral care for autists with EA. The circumstance that a survey on this topic was for the first time being carried in Germany was also rated highly.

As previously stated, our survey collected data from all of the German federal states. The study population corresponded largely with the distribution of inhabitants per federal state in terms of percent distribution. The only federal state to stand out disproportionately in this respect, with a percentage of 25.4% of the participants, was the state of Lower Saxony (Fig. 1). This finding can, however, likely be explained by the circumstance that Lower Saxony has a facility for adult autists, whose responsible body had previous contact to our research group in the context of a different study on the topic of autism and the instruction of dental medicine at university.

In our opinion, the data from our survey can be considered representative due to the fact that the participants cover all age groups, the results clearly show a higher number of P-EA in men and boys than in women and girls and the percent distribution of the participants in our study largely reflects the percent distribution of inhabitants across the federal states.

A relevant finding was that more than half of the P-EA (54.6%, *n* = 71) always need to be actively assisted in brushing their teeth (Table 3). Based on clinical experience, several of the authors would have expected this number to be considerably higher. In light of the fact that only slightly more than a tenth (13.8%, *n* = 18) of the P-EA were, in contrast, reported to always brush their teeth independently, that expectation may not be entirely unfounded. The results also showed that age was not a relevant factor in determining whether P-EA needed to be actively assisted in brushing their teeth. Statistically, the difference between the age groups of P-EA younger or older than 18 years was only 9.3% in this respect. In this context, it is important to stress that not only P-EA of all ages, but also their caregivers need to be professionally instructed in correct tooth brushing techniques and have these demonstrated to them. This had been the case for 40% of the P-EA and their caregivers (Table 5 ). Conversely, this result indicates that six out of ten P-EA in Germany had not experienced this kind of professional support. Schmidt et al. report similar findings in this respect for people with Down syndrome [20]. In addition, three-quarters (75.4%, *n* = 98) of the P-EA had not had tooth-brushing techniques practiced with them in a dental practice (Table 5).

Because intellectual disability occurs in people with Down syndrome, or can occur, as a co-morbidity in people with EA [9–11, 20], primary caregivers should be extensively trained by dental staff to take over the dental and oral care for people in these two groups as soon as possible. This recommendation is based on the likelihood that, irrespective of their age, many P-EA may lack the cognitive capacity to be able to reliably assess the need for oral and dental self-care and may also lack the necessary manual skill to perform these properly themselves. P-EA, thus, need life-long active assistance with oral self-care, perhaps even to the degree of having these procedures carried out for them. Irrespective of who performs the oral care, the P-EA themselves or caregivers, the efficiency of these procedures should be regularly assessed within the scope of dental checkups. In view of the finding that 42.3% (*n* = 55) of P-EA only started brushing their teeth at the age of one or older (Table 3), this recommendation is even more pertinent for P-EA than for others. Quite generally, the German Dental Association (BZÄK) and the National Association of Statutory Health Insurance Dentists (KZBV) recommend that a regular tooth-brushing routine should, at the latest, be introduced with the eruption of a baby’s first primary tooth, which usually occurs between the age of six and twelve months [35]. Parents or caregivers should brush their baby’s or toddler’s teeth twice daily to get the child used to an oral care routine early on and to accustom the child to the sensation of a foreign object being moving around in its mouth [36]. Moreover, a study by Zhou et al. shows that the tooth brushing behavior of six-year old children improved significantly if family members explained and demonstrated tooth brushing techniques to them, in contrast to children who were not taught how to brush [37]. Because ASD cannot be safely diagnosed before the age of three years, although behaviors typical for EA may manifest earlier, it is extremely important to introduce regular dental checkups early in a child’s life, not only to be able to recognize and address potential problems as early as possible, but also to establish a requisite level of ritual early on.

As reported by their caregivers, P-EA most frequently used manual toothbrushes for the daily care of their teeth (40.8% (*n* = 53)) (Table 3). From the open responses given by the caregivers it emerged that the vibrations and noise from electric toothbrushes often elicited intense fear in P-EA, which made tooth brushing difficult. In a recent publication, Khrautieo et al. have described that people with ASD react intensely and very sensitively to sensory stimuli. They also found that people with ASD showed greater willingness to use an electric toothbrush if this was, for example, less noisy [38]. Other caregivers reported that a manual toothbrush was used because the P-EA applied too much pressure when using an electric toothbrush, which caused problems with gum recession. In contrast, the caregivers of the 34.6% (*n* = 45) of the P-EA who used electric toothbrushes reported a better cleaning result and better reduction of gingivitis with an electric toothbrush than with a manual one. These reports reflect findings published in the literature [39, 40]. The caregivers surveyed in the present study expressed a wish for more quiet and less strongly vibrating electric toothbrushes. This wish might perhaps provide an impulse for future research focusing on specifically designed electric toothbrushes for P-EA. Only 14.6% (*n* = 19) of the P-EA used both manual and electric toothbrushes (Table 3).

As reported in numerous publications, P-EA often cling to specific routines [9, 10, 41] and disruptions of these, such as through a change of toothbrush type, may not be well tolerated. Alternating between the use of manual and electric toothbrushes is therefore not recommended. Such changes thwart the development of a routine and may possibly lead to neglect or improper execution of daily oral and dental self-care measures.

The evaluation showed that P-EA favored a variety of toothpaste options, with all types of toothpaste accepted by 50.8% (*n* = 66) of the surveyed population. Although it was striking that a quarter of the P-EA (23.8%, *n* = 31) chose their toothpaste based on flavor, this was, in particular, the case for P-EA in the age group of under 18 years old (32.7%, *n* = 18) (Table 3). In that light, the finding is unsurprising, as many non-autistic children also often describe many toothpastes as sharp or burning and prefer toothpastes with sweet or fruity flavors [42]. Very few respondents (15.4%, *n* = 20) stated that the preferred toothpaste was selected with fluoride content in mind. In the group of adult P-EA, less than 10% (*n* = 7) responded that they chose their toothpaste on the basis of fluoride content (Table 3). From a scientific point of view, the finding that fluoride content does not seem to be a relevant factor for P-FA in their choice of toothpaste - be it due of a lack of interest in specific ingredients or a lack of knowledge about the importance of fluoride in toothpastes - is noteworthy. Moreover, only every fourth autist with EA used mouth rinses or gels with fluoride (Table 4). The regular use of fluoride has been recommended ever since it became known that it could prevent caries, in 1874 [43]. Numerous published studies and guidelines thus describe the regular use of fluoridated oral care products as a key element of caries prophylaxis [43, 44, 45]. Against this backdrop, it is more striking, that the use of fluoride oral care products, such as mouth rinses or gels, was not particularly widespread among the surveyed P-EA. The use of fluoridated table salt was, was on the other hand, comparatively high, and 60.8% (*n* = 79) of the surveyed P-EA in Germany used fluoridated table salt at home (Table 4) [46, 47].

Other important parameters of dental self-care at home are the frequency and the length of tooth brushing. Our study showed that 57.7% (*n* = 75) of the P-EA brushed twice daily, and 16.9% (*n* = 22) even brushed three times daily. On the other hand, 18.5% (*n* = 24) brushed only once daily (Table 3). Thus, although the frequency of tooth brushing varied, most of the surveyed P-EA brushed their teeth twice daily. This is as often as recommended by the German guidelines for the prevention of caries in permanent teeth [43]. In contrast to the tooth-brushing frequencies found in Germany for people with Down syndrome in a study on their oral health care, a comparatively large number of P-EA brushed their teeth three times daily [20]. This finding may perhaps be attributed to the strict adherence to routines that has been described for many P-EA [9, 10, 41]. The finding that 18.5% (*n* = 24) of the P-EA, on the other hand, only brushed once daily, according to their caregivers, may be related to cognitive limitations, involuntary movements, or even downright refusal of the P-EA to cooperate [12, 13, 48, 49]. In a similar survey on people with Down syndrome, it was found that 78.3% of the surveyed people with Down syndrome brushed their teeth at least twice daily [20]. A similarly high percentage of P-EA who brushed twice daily was also found in our study. In contrast, a study on the tooth-brushing frequency in the general population showed that only around 60% of German adults brush their teeth twice daily [50].

Another German guideline recommends tooth brushing for a length of at least two minutes [51]. Only 22.3% (*n* = 29) of the P-EA in our study were capable of brushing their teeth for two minutes or more (Table 3). Possible barriers to brushing for this length of time may be poor motor skills or an inability to concentrate for so long [11, 12, 13, 48, 49]. In the survey on the oral health of people with Down syndrome mentioned above, was also found that only a third of the surveyed population was capable of brushing for longer than two minutes [20]. This finding is also backed by our clinical experience which shows that people with disability often lack the perseverance to brush their teeth for longer than two minutes.

Summary

The task of daily oral and dental self-care may be challenging, both for P-EA and their caregivers. The challenges may be due to barriers such as cognitive limitations, involuntary movements, or behaviors typical for P-EA, such as refusal or anxiety. Although not all P-AE in Germany require the same level of active assistance with their oral and dental self-care, assistance is nonetheless crucial to maintaining good oral health in most P-EA, irrespective of their age. Active assistance ought to include professional support and instruction in dental self-care. This professional service is not available yet for all P-EA and needs to be fundamentally improved. Concepts for the provision of hands-on instruction in oral and dental self-care for autists need to be developed by dentists and be given greater weight. Such concepts should include more rigorously educating caregivers about the importance of using caries-preventive fluoridated dental care products.

## Data Availability

Due to the strict European General Data Protection Regulation and the statement in the questionnaire to the study participants, no pseudonymised data will be passed on to third parties. The dataset generated from this study cannot be deposited in a public repository, because the study participant consent did not include data sharing permissions. A request for access to data for researchers who meet criteria for access to confidential data must be made to the senior author: Peter Schmidt, email: peter.schmidt@uni-wh.de, or to a representative of our Department of Special Care Dentistry, Dental School, Faculty of Health, Witten/Herdecke University, Germany and the Bundesverband Autismus Deutschland e.V. (German Association for Autism), Hamburg, Germany, too. Applicants wanting access to the dataset on which the analyses were performed must be prepared to conform to German privacy regulations. For further details, please contact, e.g., the data protection officer at the Witten/Herdecke University, Germany.

## Abbreviations

ASD: Autism spectrum disorder
P-EA: People with early childhood autism
EA: early childhood autism
